# Supplementary material for: Multiple pathways for glucose phosphate transport and utilization support growth of Cryptosporidium parvum
Source: Nat Commun. 2024 Jan 9;15:380. doi: 10.1038/s41467-024-44696-3 (PMC10774378; doi:10.1038/s41467-024-44696-3)
Supplement: Supplementary file 3 — Description of Additional Supplementary Files [file 41467_2024_44696_MOESM3_ESM.pdf]

Description of Additional Supplementary Files

File Name: Supplementary Data 1
Description: Summary of *C. parvum* proteins identified by LC-MS/MS analysis.

File Name: Supplementary Data 2
Description: Oligonucleotides and plasmids used in this study.

File Name: Supplementary Movie 1
Description: **Fluorescent intensity CpGT1-mNeon-mCh during merogony using time lapse microscopy.** CpGT1-mNeon-mCh sporozoite that invaded into HCT-8 cells and underwent the entire merogony cycle. The video is recorded from 10 min before invasion to 12 h post invasion with an interval of 1 image every 10 min. Scale bars = 5 µm.
