# Supplementary material for: Multiple pathways for glucose phosphate transport and utilization support growth of Cryptosporidium parvum
Source: Nat Commun. 2024 Jan 9;15:380. doi: 10.1038/s41467-024-44696-3 (PMC10774378; doi:10.1038/s41467-024-44696-3)
Supplement: Supplementary file 7 — Reporting Summary [file 41467_2024_44696_MOESM7_ESM.pdf]

nature portfolio

Corresponding author(s): L. David Sibley

Last updated by author(s): Nov 29, 2023

Reporting Summary

Nature Portfolio wishes to improve the reproducibility of the work that we publish. This form provides structure for consistency and transparency in reporting. For further information on Nature Portfolio policies, see our Editorial Policies and the Editorial Policy Checklist.

Statistics

For all statistical analyses, confirm that the following items are present in the figure legend, table legend, main text, or Methods section.

n/a	Confirmed	
☐	☒	The exact sample size (*n*) for each experimental group/condition, given as a discrete number and unit of measurement
☐	☒	A statement on whether measurements were taken from distinct samples or whether the same sample was measured repeatedly
☐	☒	The statistical test(s) used AND whether they are one- or two-sided<br>*Only common tests should be described solely by name; describe more complex techniques in the Methods section.*
☒	☐	A description of all covariates tested
☐	☒	A description of any assumptions or corrections, such as tests of normality and adjustment for multiple comparisons
☐	☒	A full description of the statistical parameters including central tendency (e.g. means) or other basic estimates (e.g. regression coefficient) AND variation (e.g. standard deviation) or associated estimates of uncertainty (e.g. confidence intervals)
☐	☒	For null hypothesis testing, the test statistic (e.g. *F*, *t*, *r*) with confidence intervals, effect sizes, degrees of freedom and *P* value noted<br>*Give P values as exact values whenever suitable.*
☒	☐	For Bayesian analysis, information on the choice of priors and Markov chain Monte Carlo settings
☒	☐	For hierarchical and complex designs, identification of the appropriate level for tests and full reporting of outcomes
☒	☐	Estimates of effect sizes (e.g. Cohen's *d*, Pearson's *r*), indicating how they were calculated

Our web collection on statistics for biologists contains articles on many of the points above.

Software and code

Policy information about availability of computer code

Data collection	ZEN microscope software v2.1 and v2.5; AxioVision Rel. v4.8; Image Studio v5.2; AMT Image Capture Engine v602; BioTek Gen5 v3.08; Li-Cor Odyssey imaging v3.0.
Data analysis	MUSCLE; Pfam database; ImageJ v2.0.0; ZEN microscope software v2.1; Mascot v2.7.0; Scaffold Proteome software v4.8.9; BioTek Gen5 v3.08; Image Lab v6.1; Volocity v6.3; GraphPad Prism v9.

For manuscripts utilizing custom algorithms or software that are central to the research but not yet described in published literature, software must be made available to editors and reviewers. We strongly encourage code deposition in a community repository (e.g. GitHub). See the Nature Portfolio guidelines for submitting code & software for further information.

Data

Policy information about availability of data

All manuscripts must include a data availability statement. This statement should provide the following information, where applicable:

- Accession codes, unique identifiers, or web links for publicly available datasets
- A description of any restrictions on data availability
- For clinical datasets or third party data, please ensure that the statement adheres to our policy

All of the data are found in the main figures or supplementary information. Source data are provided with this manuscript.

Human research participants

Policy information about studies involving human research participants and Sex and Gender in Research.

Reporting on sex and gender	N/A
Population characteristics	N/A
Recruitment	N/A
Ethics oversight	N/A

Note that full information on the approval of the study protocol must also be provided in the manuscript.

Field-specific reporting

Please select the one below that is the best fit for your research. If you are not sure, read the appropriate sections before making your selection.

☒ Life sciences ☐ Behavioural & social sciences ☐ Ecological, evolutionary & environmental sciences

For a reference copy of the document with all sections, see nature.com/documents/nr-reporting-summary-flat.pdf

Life sciences study design

All studies must disclose on these points even when the disclosure is negative.

Sample size	We did not have any data for different estimates of variability needed for power or sample size analysis. The number of data points collected from each sample was based on the minimum required to perform statistical comparisons. For within group sizes, minimal group sizes were n = 3.
Data exclusions	None of the data points were excluded for the analysis.
Replication	All experiments were repeated at least 2 or 3 times with 2-3 biological replicates. All results were successfully replicated.<br>In vivo experiment, mouse numbers (n = 6 or 7 in each group from two independent experiments) were based on our previously published work and elsewhere in the literature.
Randomization	The mice used in measuring parasite growth study of TAG or KO strain were randomly divided into two groups. Mice were number coded and randomly assigned to a group prior to the infection. Randomization was not applied in other experiments since the groups are defined a priori (i.e. control vs knockout) and covariants are not applicable. Where different parasite strains were used to infect host cells, they were randomly assigned to different wells within the experimental design.
Blinding	We did not blind the samples. In all of the experiments, the readouts of the assays are quantitative and not subject to investigator interpretation. Variation on the outcome was addressed using replicates.

Reporting for specific materials, systems and methods

We require information from authors about some types of materials, experimental systems and methods used in many studies. Here, indicate whether each material, system or method listed is relevant to your study. If you are not sure if a list item applies to your research, read the appropriate section before selecting a response.

Materials & experimental systems

n/a	Involved in the study	
☐	☒	Antibodies
☐	☒	Eukaryotic cell lines
☒	☐	Palaeontology and archaeology
☐	☒	Animals and other organisms
☒	☐	Clinical data
☒	☐	Dual use research of concern

Methods

n/a	Involved in the study	
☒	☐	ChIP-seq
☒	☐	Flow cytometry
☒	☐	MRI-based neuroimaging

Antibodies

Antibodies used	Primary antibodies used in this study: rat anti-HA (Roche, mAB 3F10, Cat# 11867423001, diluted 1:1000); rabbit anti-HA (Invitrogen, pAB SG77, Cat# 71-5500, diluted 1:1000); rabbit anti-HA antibody (Sigma, pAB, Cat# H6908, diluted 1:1000); mouse anti-Ty (in house hybridoma, mAB clone BB2, diluted 1:500); mouse 1B5 (in house hybridoma, mAB, diluted 1:500); mouse 1E12 (in house hybirdoma, mAB, diluted 1:500); mouse 4D8 (in house hybridoma, mAB, diluted 1:20); rabbit PanCp (in house, pAB, diluted 1:2000); rat PanCp (in house, pAB, diluted 1:2000).<br><br>Secondary antibodies used in this study:<br>Alexa Fluor 488 Goat anti-rat IgG (H+L) (Thermo Fisher, Cat#A-11006, diluted 1:1000); Alexa Fluor 568 Goat anti-mouse IgG (H+L) (Thermo Fisher, Cat#A-11004, diluted 1:1000); Alexa Fluor 568 Goat anti-mouse IgM (Heavy chain) (Thermo Fisher, Cat#A-21043, diluted 1:1000); Alexa Fluor 647 Goat anti-rabbit IgG (H+L) (Thermo Fisher, Cat#A-11011, diluted 1:1000); Alexa Fluor 568 conjugated Streptavidin (Thermo Fisher, Cat#S11226, diluted 1:1000); IRDye 800CW Streptavidin (LI-COR Biosciences, Cat#926-32230, diluted 1:2000); Alexa Fluor 488 Goat anti-rabbit IgG (H+L) (Thermo Fisher, Cat#A-11034, diluted 1:1000); Alexa Fluor 647 goat anti-rat IgG (H+L) (Thermo Fisher, Cat#A-21247, diluted 1:1000); 18nm colloidal gold conjugated goat anti-Rabbit IgG (H+L) (Jackson ImmunoResearch, Cat# 111-215-144, diluted 1:20).
Validation	Rat anti-HA (3F10) (https://www.sigmaaldrich.cn/CN/en/product/roche/12158167001) was validated by western blot and ELISA in the product description.<br><br>Rabbit anti-HA (SG77) (https://www.thermofisher.cn/cn/en/antibody/product/HA-Tag-Antibody-clone-SG77-Polyclonal/71-5500) was validated by western blot and immunofluorescence in the product description.<br><br>Rabbit anti-HA antibody (pAB)(https://www.sigmaaldrich.cn/CN/en/product/sigma/h6908) was validated by western blot, immunofluorescence and immunoprecipitation in the product description.<br><br>Mouse 1B5, 1E12 and 4D8, rabbit PanCp and rat PanCp: in house hybridoma was originally generated from: G. Wilke, S. Ravindran, L. Funkhouser-Jones, J. Barks, Q. Wang, K. L. VanDussen, et al.Monoclonal antibodies to intracellular stages of Cryptosporidium parvum define life cycle progression in vitro. mSphere. 3(3): e00124-18, (2018). They were validated in the lab by testing staining C. parvum different life stages.<br><br>Mouse anti-Ty: in house hybridoma was originally obtained from: Bastin, P., Bagherzadeh, A., Matthews, K. R. & Gull, K. A novel epitope tag system to study protein targeting and organelle biogenesis in Trypanosoma brucei. Mol. Biochem. Parasitol. 77, (1996). It was validated in the lab by testing against protein standards bearing this epitope tag.

Eukaryotic cell lines

Policy information about cell lines and Sex and Gender in Research

Cell line source(s)	Human ileocecal adenocarcinoma cells (HCT-8; ATCC CCL-244)
Authentication	The cells were tested by short tandem repeat analysis by the Genome Engineering and Stem Cell Center at Washington University School of Medicine in St. Louis.
Mycoplasma contamination	All cell lines and cultures were tested for Mycoplasma contamination using the e-Myco plus mycoplasma PCR detection kit following the manufacturer's manual (Boca Scientific).
Commonly misidentified lines<br>(See ICLAC register)	No commonly misidentified cell lines were used in the study.

Animals and other research organisms

Policy information about studies involving animals; ARRIVE guidelines recommended for reporting animal research, and Sex and Gender in Research

Laboratory animals	Ifng-/- mice (referred to as GKO) (002287; Jackson Laboratories), and Nod scid gamma mice (referred to as NSG) (005557; Jackson Laboratories) were bred in-house in a specific-pathogen-free animal facility. The room housing mice were maintained at humidity 30-70%, temperature 20-26°C, on a 12:12 light:dark cycle. Mice were co-housed with siblings of the same sex throughout the experiments.
Wild animals	No wild animals were used in the study.
Reporting on sex	Male and female mice between 8 and 10 weeks of age were randomly allocated to each experimental group. The prior studies have not shown a sex difference in C. parvum infectivity. We did not test the influence of sex on outcome due to small sample size.<br>In Figure 3a, 3 male of NSG mice and 3 female of NSG mice (n = 6, from two independent experiments) used for measuring parasite burden of CpGT1 TAG strain, and 3 male of NSG mice and 3 female of NSG mice (n = 6, from two independent experiments) used for measuring parasite burden of CpGT1 KO strain. In Figure 3b, 3 male of NSG mice and 3 female of NSG mice (n = 6, from two independent experiments) used for measuring parasite burden of CpGT2 TAG strain, 4 male of NSG mice and 3 female of NSG mice (n = 7, from two independent experiments) used for measuring parasite burden of CpGT2 KO strain. In figure 3e, 3 female of NSG mice (n = 3, from a single experiment) used for measuring parasite burden of CpHK KO strain from one experiment.<br>In Supplementary Fig. 7b, 3 male of GKO mice and 3 female of GKO mice (n = 6, from two independent experiments) used for

	selection of Cp aldolase KO strain. In Supplementary Fig. 7d, 3 female of GKO mice (n = 3, from a single experiment) used for selection of CpGP KO strain.
Field-collected samples	No field collected samples were used in the study.
Ethics oversight	Animals that became non-ambulatory during the course of infection were humanely euthanized in a SMARTBOX Auto CO2 euthanasia chamber. Animal studies on mice were approved by the Institutional Animal Studies Committee (School of Medicine, Washington University in St. Louis).

Note that full information on the approval of the study protocol must also be provided in the manuscript.
